# Whole-Genome Sequence of Fish-Pathogenic *Mycobacterium* sp. Strain 012931, Isolated from Yellowtail (*Seriola quinqueradiata*)

**DOI:** 10.1128/genomeA.00534-13

**Published:** 2013-08-08

**Authors:** Satoru Kurokawa, Jun Kabayama, Seong Won Nho, Seong Don Hwang, Jun-ichi Hikima, Tae Sung Jung, Hidehiro Kondo, Ikuo Hirono, Haruko Takeyama, Takashi Aoki

**Affiliations:** Animal Health Department of Research and Development, Agricultural and Veterinary Division, Meiji Seika Pharma, Tokyo, Japana; Aquatic Biotechnology Center of the WCU Project, College of Veterinary Medicine, Gyeongsang National University, Gyeongnam, South Koreab; Institute of Marine Industry, Department of Marine Biology and Aquaculture, College of Marine Science, Gyeongsang National University, Tongyeong, South Koreac; Laboratory of Genome Science, Tokyo University of Marine Science and Technology, Tokyo, Japand; Department of Life Science and Medical Bioscience, Waseda University, Tokyo, Japane; Consolidated Research Institute for Advanced Science and Medical Care, Waseda University, Tokyo, Japanf

## Abstract

The genus *Mycobacterium* comprises a large number of well-characterized species, several of which are human and animal pathogens. Here, we report the whole-genome sequence of *Mycobacterium* sp. strain 012931, a fish pathogen responsible for huge losses in aquaculture farms in Japan. The strain was isolated from a marine fish, yellowtail (*Seriola quinqueradiata*).

## GENOME ANNOUNCEMENT

Mycobacteria are members of the family *Mycobacteriaceae* in the order *Actinomycetales*. They are pleomorphic, Gram-positive, acid fast, nonmotile, nonsporulating, and rod shaped (1). The genus *Mycobacterium* currently contains 148 recognized species, including *M. marinum*, *M. pseudoshottsii*, and an unidentified *Mycobacterium* sp., which are the mostly commonly identified bacterial fish pathogens (2, 3). Some are also zoonotic, causing cellulitis, skin tuberculosis, and foreign body reactions in humans (4).

In 1985, an epidemic caused by an unidentified *Mycobacterium* sp. spread in an aquaculture farm of yellowtail (*Seriola quinqueradiata*) in Sukumo Bay, Japan (5), and caused hemorrhagic ascites and hypertrophy of the spleen and kidney with tubercles and visceral adhesions (5). Later, the same *Mycobacterium* sp. was shown to affect cultured striped jack (*Pseudogaranx dentex*) and amberjack (*Seriola dumerili*) (6). These bacterial strains are responsible for declines in fish production and, ultimately, huge losses in the Japanese aquaculture industry. Existing chemotherapeutic agents, including rifampicin, streptomycin, and erythromycin, can provide limited protection against the unidentified *Mycobacterium* sp., but this strain can proliferate after antibiotic treatment (7). Therefore, an effective vaccine against the disease is needed.

*Mycobacterium* sp. strain 012931, previously reported as the TUMSAT-Msp001 strain (3), causes the same disease in yellowtail as described above. To clarify and understand genomic features of this unknown strain, we conducted whole-genome sequencing of *Mycobacterium* sp. strain 012931 and compared the sequence to two genome sequences of *M. marinum* fish isolates recently reported (MB2, GenBank accession no. ANPM00000000, and Europe, ANPL00000000) (8).

We report the whole-genome sequence of *Mycobacterium* sp. 012931 isolated from yellowtail (*Seriola quinqueradiata*) in Japan. Genomic DNA of *Mycobacterium* sp. 012931 was extracted by a previously described method (9) and sequenced by using a Roche GS-FLX 454 platform manufactured by TaKaRa Bio, Inc (Otsu, Japan). The reads were assembled with Newbler software (version 2.6). Coding sequences (CDS) of *Mycobacterium* sp. 012931 were predicted and annotated by use of the RAST server (10). The conserved protein domain analysis was performed using BLASTP (11). tRNA sequences were identified by tRNA scan-SE (12).

*Mycobacterium* sp. 012931 has a double-strand DNA genome of 5,759,364 bp with a 65.7% GC content and is represented by a single scaffold. The genome contains 5,454 CDS classified into 26 subsystems, of which 2,249 CDS were classified as unknown genes or genes of hypothetical function. The genome contains 50 tRNA sequences. Among the 5,454 CDS, 478 genes were categorized as encoding amino acids and derivatives, 445 genes as encoding cofactors, vitamins, prosthetic groups, and pigments, and 405 genes as encoding fatty acids, lipids, and isoprenoids and 477 genes were associated with carbohydrates. Furthermore, a total of 171 genes were found to be associated with virulence, disease, and defense. *Mycobacterium* sp. 012931 and *M. marinum* strains MB2 and Europe all have a PE/PPE gene cluster, which contains genes related to virulence, disease, and defense subsystems. A comparison of these clusters in the three strains revealed numerous mutations and substitutions.

### Nucleotide sequence accession number.

The complete genome sequence of *Mycobacterium* sp. 012931 was deposited in the GenBank database under accession number AOPX00000000.
